# Omega-3 fatty acids, brain health and the menopause

**DOI:** 10.1177/20533691251341701

**Published:** 2025-05-30

**Authors:** Anne Marie Minihane

**Affiliations:** 1 Norwich Medical School, 6106 University of East Anglia (UEA), Norwich, UK; 2 Norwich Institute of Healthy Ageing (NIHA), Centre for Lifespan Health, 6106 UEA, Norwich, UK

**Keywords:** Sleep, vasomotor symptoms, brain fog, memory, anxiety, docosahexaenoic (DHA), eicosapentaenoic acid (EPA)

## Abstract

The menopausal transition is associated with vasomotor symptoms, disrupted sleep, transient cognitive deficits and changes in mood and anxiety levels, underpinned by declining and erratic estrogen availability in the brain. Relative to other tissues the brain is enriched in the omega-3 fatty acid, docosahexaenoic (DHA), with well-defined neurophysiological roles for both eicosapentaenoic acid (EPA) and DHA. Substantial preclinical and epidemiological evidence along with accumulating randomised controlled trial (RCT) data indicates that an increase in EPA and DHA intake and status is associated with improved brain function. In this narrative review, the role of EPA and DHA in the menopausal transition (MT) is considered. The evidence, although relatively sparse, is indicative of benefit, with future RCTs needed to establish dose–response relationships and when it is most beneficial to intervene. Although research is at a relatively early stage, the MT is emerging as a critical window of intervention opportunity not only to support MT well-being but also lifelong health in women.

## Introduction

Over 100 different menopausal symptoms^1^ have been associated with the menopausal transition (MT), which we define as perimenopause and up to 5 years postmenopause, which are directly or indirectly related to declining estrogen and progestogen and increases in follicle stimulating hormone. Over 80% of women suffer menopausal symptoms^2^ with an estimated 10% leaving the work force in the UK as a result.^3^ A major cause of this menopausal-associated work force attrition and a deterioration in quality of life^4^ is due to MT-associated vasomotor symptoms and a cognitive phenotype termed brain fog characterised by deficits in attention, memory, verbal fluency and executive function, along with changes in mood and anxiety levels.^5–8^ Although currently unknown, as longitudinal studies examining the impact of MT cognitive status and cognitive health in older age are lacking, MT brain fog may be a determinant of dementia risk.^9^ The timing of MT coincides with and may represent the early prodromal dementia phase in some women, which is known to last typically 20–30y.^10^ Therefore, MT is a critical window of intervention opportunity not only for optimising brain health during MT but also to potentially promote long-term brain and overall health.

Brain tissue is particularly enriched in the omega-3 fatty acid, docosahexaenoic acid (DHA), with well-defined structural and functional roles. Here, we summarise existing literature on the effect of omega-3 intake and status on MT vasomotor symptoms and brain health and identify future research needs.

## Omega-3 fatty acids explained, along with recommended and actual intakes

**Figure 1.**
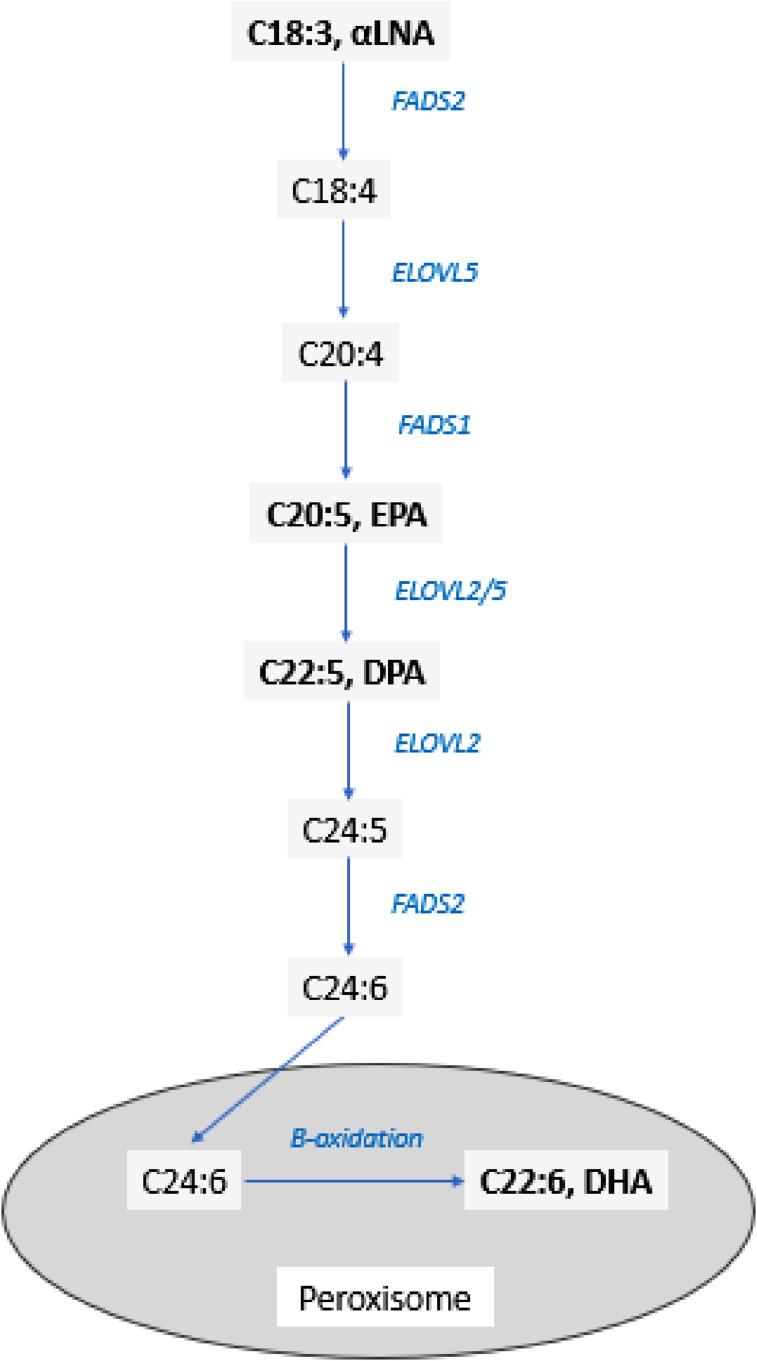
Pathway of conversion of α-linolenic acid (αLNA) to EPA and DHA. ALA, alpha-linolenic acid; EPA, eicosapentaenoic acid; DPA, docosapentaenoic acid; DHA, docosahexaenoic acid; FADS1, fatty acid desaturase 1/delta-5 desaturase/Δ5-desaturase; FADS2, fatty acid desaturase 2/delta-6 desaturase/Δ6-desaturase; ELOVL, fatty acid elongase.

The predominant dietary omega-3 fatty acids are alpha-linolenic acids (αLNA, C18:3), and the long chain omega-3 fatty acids (LCO3FA), eicosapentaenoic acid (EPA, C20:5) and DHA (C22:6). αLNA is termed an essential fatty acid, which cannot be synthesised by mammalian tissue, with important dietary sources including rapeseed, soybean and flaxseed oils, along with walnuts and other select nuts and seeds, soybeans and green leafy vegetables. Although EPA and to a lesser extend DHA can be synthesised from αLNA, and are therefore not strictly essential, bioconversion (Figure 1)^11^ in humans is limited with conversion rates of 0·2–6 % for EPA and <0 · 1 % for DHA in males and post-menopausal females.^12^

Premenopausal are several-fold more efficient at EPA and DHA synthesis,^12^ with estimated net fractional α[^13^C]LNA inter-conversion to EPA of 21% and DHA of 9% over 21 days reported in healthy women with a mean age of 28 years.^13^ This enhanced ability to synthesise EPA/DHA has an evolutionary basis, serving to provide EPA/DHA to the developing foetus and newborn during pregnancy and lactation.^14^

Estrogen is known to decrease αLNA oxidation^12^ and increase the hepatic expressions of FADS2 (the rate-limiting enzyme in DHA synthesis), ELOVL2 and ELOVL5, thereby increasing the conversion of αLNA into DHA.^15,16^ As women transition through the menopause this enhanced ability to synthesise LCO3FA is lost, with HRT use associated with increased DHA but not EPA status,^17^ likely due to an upregulation of ELOVL2^15,17^(Figure 1).

Given the low endogenous synthesis of EPA and DHA, in the UK, the advice is to eat two portions of fish per week (140 g each), one of which should be oily, with a recommended EPA + DHA intake of greater than 450 mg per day.^18^ This is in line with the International Society for the Study of Fatty Acid and Lipids (ISSFAL)^19^ and the American Heart Association (AHA)^20^ recommendations of at least 500 mg per day for the general population. Typically, an additional 200–300 mg of DHA or EPA + DHA is recommended during pregnancy and lactation.^21^ Although EPA and DHA intake and status are recognised to be important to health throughout the life course from conception into older age,^22^ intakes are substantially lower with an average population intake of 200–250 mg/day, and the majority consuming <50 mg EPA + DHA/day,^23^ with the average heavily skewed by the approximate 25% of the population who are oily fish (almost exclusive dietary EPA/DHA source) consumers.^24^ Oily fish consumption also demonstrates a strong socio-economic gradient with affluent groups 2.4–4.0 times more likely to eat oily fish than the least affluent groups in the UK.^25^ Also, in the UK oily fish consumption tends to increase with age.^24^ Fish oil, omega-3 or microalgal oil supplements represent an alternative EPA and DHA source to oily fish, although oily fish is thought to be a superior source given that it is also rich in several other nutrients important to human health, for example, essential amino acids, vitamin B12, vitamin D, iodine and selenium.

Interestingly, looking at estrogen, menopause and LCO3FA status in reverse, there is evidence that LCO3FA may increase ovarian reserve and increase the age of natural menopause and reduce the risk of early menopause,^26^ with a number of mechanisms proposed such as, an anti-inflammatory impact and a lowering of oxidative stress which reduce follicular atresia (loss of eggs), and improved blood flow and mitochondrial function which support ovarian function.^27,28^

## EPA, DHA and brain health

**Figure 2.**
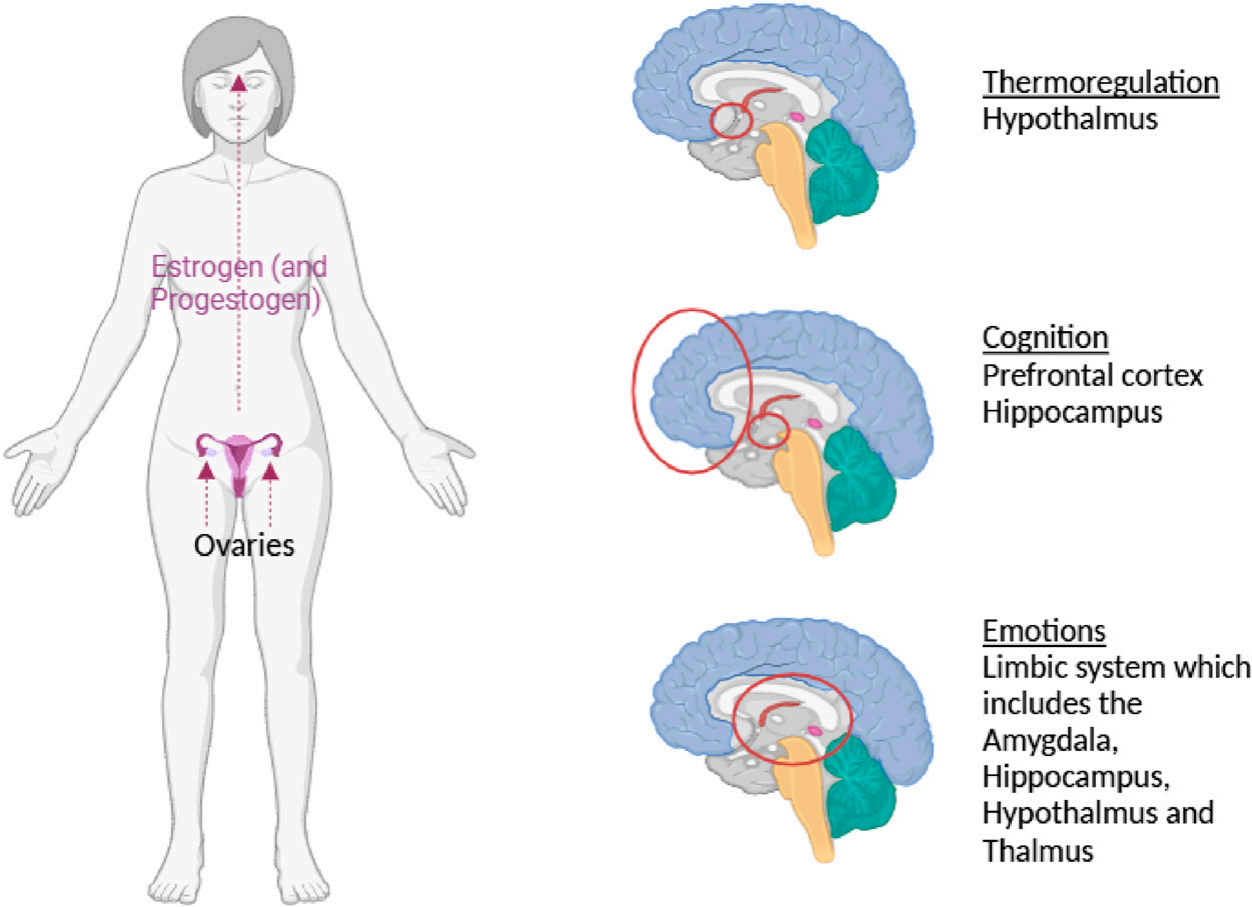
Overview of the role of estrogen on key brain regions.

A high consumption and tissue status of EPA and DHA is associated with lower total mortality.^29^ In addition to reduced cardiovascular mortality, a large body of preclinical and epidemiological evidence indicates that this reduced mortality may be partly due to the positive impact of LCO3FAs on neuropathology, cognitive decline and risk of dementia and depression.^29–32^ Part of the brain health benefits of EPA and DHA is likely due to improved cardiovascular health including enhanced blood–brain barrier (BBB) function and cerebral perfusion.^33^ Additionally, DHA is a major structural lipid in the brain, and in particular in neurones, contributing to membrane fluidity and functionality. It constitutes 15% of brain total lipids compared to <5% in most other major organs, with select synaptic lipids having up to 40% DHA.^34^ Although overall brain EPA levels are low and as a result it has been somewhat ignored when it comes to brain health, EPA is efficiently taken up into the brain via the BBB and is enriched in microglial.^35^ DHA and EPA have been shown to have numerous key functional roles in the brain (Figure 2) important in cognition such as the prefrontal cortex, hippocampus and entorhinal cortex and the hypothalamus responsible for thermoregulation and the vasomotor symptoms of menopause, such as the regulation of synaptic plasticity, neurogenesis, glucose utilisation and mitochondria function, neuroinflammation, neurotransmitter metabolism, myelination, BBB tight junction integrity and β-amyloid and TAU metabolism (two key pathological proteins in Alzheimer’s disease).^30,36^

Randomised controlled trial evidence is generally supportive of a positive impact of EPA and DHA on depressive symptoms.^37–39^ The RCT evidence for cognitive benefits is more mixed.^40,41^ Most are secondary prevention interventions, conducted in individuals with significant cognitive impairment,^31^ with often small sample sizes, short intervention periods, cognition as a secondary outcome and little consideration given to habitual EPA + DHA status. In contrast in young healthy adults with low habitual fish intake, 1.4 g EPA + DHA per day for 6 months improved both episodic and working memory.^42^ Traditionally RCTs focussed on brain health have intervened with DHA-rich supplements, with Pattan and colleagues demonstrating that EPA but not DHA improved global cognition over 6 months in healthy adults.^43^ Longer-term RCTs in healthy or prodromal participants are needed to fully establish the efficacy of EPA + DHA in promoting cognition and reducing life-long dementia risk.

Interestingly dementia risk is twice as high in women, which is only in part explained by a longer life, with higher age-standardised rates in women of all ages over the age of 65 years.^44^ This may in part be due to brain EPA + DHA depletion with the observation of lower DHA with age in a rodent model which was more evident in females, and in particular in *APOE4* carriers (the main genetic risk factor for cognitive decline and AD).^45^ Menopause is associated with a lower brain EPA and DHA in rodents,^46^ which is mitigated by increased EPA and DHA intake.^47^

Therefore, EPA and DHA supplementation offers significant potential as a therapy for MT-associated vasomotor symptoms, brain fog, low mood and anxiety.

### Hormonal changes during menopause

During the reproductive years estrogen is mainly produced by the granulosa cells in the ovaries (Figure 2), with small amounts also produced by other organs such as the adipose tissue, adrenal glands, bone, breast, skin, liver and brain. In addition to its primary role in sexual maturation and reproductive function, estrogen modulates cardiovascular, musculoskeletal, immune function and brain function which is the focus of the current review. Estrogen receptors including estrogen receptor-alpha (ERα), estrogen receptor-beta (ERβ) and G-protein coupled estrogen receptor 1 (GPER) are expressed throughout the brain, with multiple estrogen responsive pathways, for example, in the amygdala, prefrontal cortex and hypothalamus affecting mental health, cognition and thermoregulation, respectively. During the MT, the production of estrogen (and progesterone) declines significantly which underpins the diverse array of menopausal-associated brain symptoms such as anxiety, depression, brain fog and vasomotor symptoms.

### Omega-3 fatty acids and vasomotor symptoms (VSM) including sleep

Vasomotor symptoms (VMS) are characterised by hot flashes (HF) and night sweats in the head, neck, chest and upper back and are typically associated with disrupted sleep.

Although HRT remains the prevailing strategy for mitigating VMS, it is unsuitable for many women either based on side-effects or contraindication because of health status such as incident breast cancer. Therefore, alternative approaches are needed.

A small (*n* = 393) cross-sectional analysis reported that total omega-3 PUFA and EPA intake was associated with a lower incidence of somatic symptoms (which includes HF and sleep disturbances) on the Menopause Rating Scale (MRS) questionnaire.^48^ Only limited RCT evidence is available with mixed findings. In the largest of these the MsFLASH study, 12 weeks of EPA + DHA supplementation had no effect on VSM frequency, VSM ‘bother’ or sleep quality, although the results are difficult to interpret as the study had a 3 × 2 factorial design with approximately two-thirds of participants also assigned to an ‘exercise’ intervention.^49^ In contrast, Lucas et al., reported that 8 weeks of supplementation with EPA reduced HF frequency and improved the HF score^50^ with Purzand et al., observing that EPA + DHA improved somatic and overall MRS score.^51^ In a 2023 systematic review of the limited RCT evidence, the combined analysis of the five and three RCTs available did not provide substantial evidence of a benefit of EPA and DHA in improving VSM and sleep quality, respectively.^52^ However, given the scarcity of the evidence it is far from conclusive, with future longer-term adequately powered larger MT and/or postmenopausal studies needed.

Interestingly interventions^53,54^ and a systematic review and meta-analysis,^55^ which have looked at the impact of omega-3 PUFA on sleep quantity and quality in a general health population, highlight the potential of this intervention to improve sleep efficiency in menopause.

Also, two studies have shown that using vitamin E and omega-3 in combination reduces HF and^56^ indicates the potential of a co-supplementation approach to mitigate VSM and sleep disturbances. No RCT to date has specifically focussed on perimenopause (which arguably is the most responsive window of opportunity for intervention), which represents a research gap.

### Omega-3 fatty acids, mental health and cognition in menopause

The MT is often associated with a worsening of mood and anxiety. Women are twice as likely to experience major depression than men.^57^ Evidence suggests that some women might experience an increased risk for developing depression during ‘windows of vulnerability’, that is, when exposed to intense hormone fluctuations, such as the MT. The impact of EPA and DHA on mental health during MT shows promise but the evidence is as yet not conclusive.^58^ In a 2022 systematic review, six animal studies, four human RCTs, one human open-label trial and six human observational studies were considered, with the authors concluding that ‘most studies reported relieved depressive symptoms in relation to omega-3 PUFA intake’.^59^ In an analysis of baseline data (*n* = 3053) from the Study of Women’s Health Across the Nation (SWAN), omega-3 fatty acid intake was inversely dose-dependently associated with depressive symptoms in perimenopause.^60^ In a trial which included 40–55 year old women with psychological distress, although no effect of EPA supplementation for 8 weeks was evident in the group as a whole, an improvement in depression symptoms was observed in those participants without major depression.^61^

Much of the cognitive benefits of omega-3 intake in MT and postmenopausal come from rodent studies.^59^ In ovariectomised rats, a DHA-rich tuna oil was associated with improved memory and anti-oxidant capacity and decreased inflammation,^62^ whereas in a model of natural human menopause, DHA-rich fish oil mitigated the decline in recognition memory associated with menopause, and in particular in *APOE4* animals.^47^ This was associated with a restoration of brain DHA levels and changes in the expression of key brain genes involved in glucose utilisation and BBB function. RCTs which investigate the impact of omega-3 supplementation during MT and underlying physiological mechanisms are much needed.

Part of the benefits of omega-3 fatty acids on brain health is likely to be due to improved systemic cardiovascular health, cardiovascular health and brain health,^63^ with EPA + DHA intake associated with improved vascular function^64^ and lower blood pressure^65^ and circulating TG.^65,66^

## Conclusion

Estrogen receptors are expressed throughout the brain with ongoing research characterising the impact of estrogen decline during the MT on brain function, including VSM symptoms, cognition and emotional well-being. The omega-3 fatty acids EPA and DHA are integral to brain health, with their benefits in supporting cognitive health and reducing the risk of depression and anxiety in adulthood evident. Although trial evidence demonstrating the impact of EPA and DHA supplement on VSM, brain fog, mood and anxiety during MT is as yet spare, it does demonstrate promise, with further research needed to fully establish the optimal timing of intervention during MT, dose and ratio of EPA and DHA and effective co-supplementation regimes. In addition to MT health, it is highly likely that nutrition and eating behaviour during this critical period is a major determinant of health and risk of disease in later life in women.
